# Deterministic Current Induced Magnetic Switching Without External Field using Giant Spin Hall Effect of β-W

**DOI:** 10.1038/s41598-018-26586-z

**Published:** 2018-05-25

**Authors:** Wenzhe Chen, Lijuan Qian, Gang Xiao

**Affiliations:** Department of Physics, Brown University, Providence, Rhode Island 02912 USA

## Abstract

Giant spin Hall effect (GSHE) has received significant attention for its potential in future spintronic applications. Spin current via GSHE-based thin films provides an effective and promising means to manipulate magnetization. However, an external in-plane magnetic field is required to consistently switch the perpendicular magnetic moment. We present an approach to realize field-free deterministic perpendicular magnetic switching with a new structure of FM/NM/FM. Our method takes advantage of the large spin Hall angle of transition metal β-W, so that the critical switching current density is only on the order of 10^6^A/cm^2^ in the absence of magnetic field.

## Introduction

In recent years, electrical manipulation of magnetism using giant spin Hall effect (GSHE) has been of great interest to the development of ultrafast and reliable spintronic logic and memory devices^1–4^. Studies^2,4–11^ have revealed that transition metals such as Hf, Pt, β-Ta and β-W exhibit very large spin Hall angles (SHAs) due to their strong spin-orbit coupling. With a large SHA, a charge current is efficiently converted into a spin current, whose spin-transfer torque can be used to manipulate the magnetization vector of a neighboring ferromagnetic metal film (FM) or a particle element. In spintronic devices, the magnetization vector is used as a memory agent, a logic state, or a field sensing vehicle. However, to deterministically control the perpendicular magnetization vector requires an in-plane external biasing field on the FM. The generation of this biasing field either complicates the device structure (*e.g*., using a local biasing magnet) or consumes energy (*e.g*., using electrical current to generate a Faraday field). For these reasons, spintronics research community actively pursues the achievement of a field-free deterministic magnetic switching.

Currently, there are a few methods to generate field-free switching. One method^12^ is to make a wedge-shaped GSHE layer to obtain so-called lateral structure asymmetry, which is not easy to implement in practical manufacturing. The other method^13–17^ is to replace the GSHE transition metal with an antiferromagnetic metallic layer (AFM) and form an AFM/FM structure. The interfacial exchange field acts as the biasing field, but the exchange bias is often found to be insufficient, leading to partial field-free magnetic switching^17^. Furthermore, the critical switching current density is large, due to the relatively small SHA typically found in an AFM^18^.

In this article, we introduce a method of field-free switching in a structure of FM/NM/FM, where the central NM is a GSHE non-magnetic metallic layer. The bottom FM with an in-plane magnetization vector exerts a biasing field on the top FM which has an out-of-plane and switchable magnetization vector. We demonstrate complete, deterministic, and reliable field-free switching, as well as a low critical switching current density. We used β-W as the NM which has the largest GSHE discovered^8,19^, however other transition metals with GSHE can also be used in our design^2,4,5,11^. The key to our success is an elaborate magnetic annealing scheme to develop the orthogonal magnetization states between the two FM layers.

## Sample Preparation and Characterization

**Figure 1:**
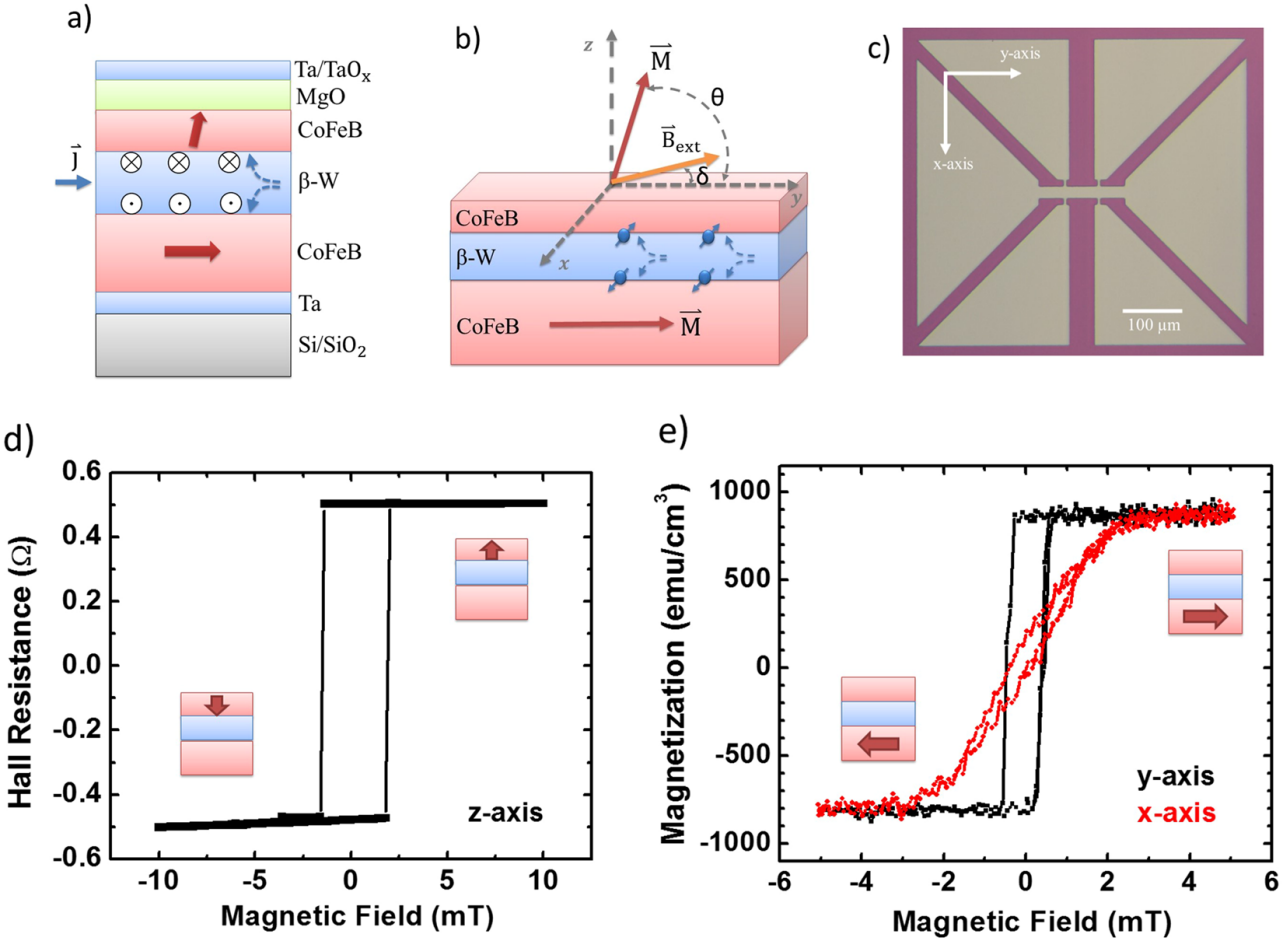
The structure and characterization of multilayer sample Si/SiO_2_/Ta(1.0)/CoFeB(3.0)/β-W(x)/CoFeB(1.0)/MgO(1.6)/Ta(1.0). (**a**) Cross-section view of our multilayer sample. The red arrows in CoFeB layers are magnetization’s easy axis. The out-of-plane vectors on two β-W/CoFeB interfaces show the accumulated spin as a result of the β-W’s giant spin Hall Effect. (**b**) Schematic of Hall measurement setup. θ is the angle between the top layer’s magnetization and y-axis and δ is the angle between the external magnetic field and y-axis. (**c**) Optical microscopic image of the fabricated Hall bar device. (**d**) Anomalous Hall resistance with perpendicular (z-axis) magnetic field. The high/low Hall resistance refer to the spin-up/spin-down state (shown as red arrow) of top CoFeB layer. (**e**) Magnetization hysteresis loops within the magnetic field of 5 mT along x-axis (red curve) and y-axis (black curve).

We prepared our samples by depositing stacks of multilayered films on thermally oxidized silicon wafers using the technique of high vacuum magnetron sputtering. As shown schematically in Fig. 1(a), the bottom-up multilayer is sequenced as: Si/SiO_2_/Ta(1.0)/CoFeB(3.0)/β-W(x)/CoFeB(1.0)/MgO(1.6)/Ta(1.0), (numbers inside parenthesis referring to thicknesses with units in nanometer (nm) and CoFeB standing for ferromagnetic alloy Co_40_Fe_40_B_20_). Each sample is specified by the thickness (x) of the β-W GSHE layer. The bottom Ta layer acts as a seed layer to promote the growth of the samples. The 3-nm-thick CoFeB with its in-plane magnetization is used to provide a biasing field, mediated through the β-W layer, to the top 1-nm-thick CoFeB layer with an out-of-the-plane magnetization. The perpendicular magnetic anisotropy (PMA) is facilitated by the neighboring MgO layer. The capping layer Ta is partially oxidized due to exposure to the atmosphere and used to passivate the samples from oxidation. All the samples were annealed in a high vacuum chamber (~10^−6^ Torr) at a temperature of ~280 °C for 1 hour with a magnetic field of ~0.42 T applied in the plane of the stacks. After sample annealing, we measured the resistance of the whole stack to confirm the β-phase of W layer. Using the rule of parallel resistance in multilayers, we estimate that the resistivity of the W-layer is in a range of 191.1 to 301.8 µΩ-cm for samples with x in the range of 3.6 to 7.0 nm. The typical resistivity values^6,20^ for α-W and β-W are 30~40 µΩ-cm and 100~300 µΩ-cm, respectively. Hence, all our samples contain the β-phase W, which serve as the GSHE solid. We also used X-ray diffraction to ascertain the structure of β-W formation.

Figure 1(b) shows the key component layers of CoFeB(3.0)/β-W(x)/CoFeB(1.0) and the associated magnetizations states in a Hall-effect measurement configuration within the illustrated Cartesian coordinate system (x-y-z), with the in-plane y-axis along the same direction of the annealing magnetic field. An external magnetic field (${\mathop{{\rm{B}}}\limits^{\rightharpoonup }}_{{\rm{ext}}}$) is applied within the y-z plane at an angle (δ) with respect to the y-axis. An electrical current applied along the y-axis within the β-W layer is converted into a spin current along the z-axis, with an efficiency proportional to the spin Hall angle (Θ) of β-W. The spin current creates a spin-transfer-torque onto the magnetization vector of the top PMA CoFeB(1.0) layer. From here on, we will present our measurement results on a typical sample with x = 5.4 nm for the β-W layer, unless specified otherwise.

We confirmed the natural spin states of Fig. 1(b) using the following experiment. Figure 1(d) shows the anomalous Hall-effect resistance of the sample as a function of a cycling perpendicular magnetic field along z-axis within ±10 mT. The observed perfect square loop indicates the robust PMA of top 1-nm-thick CoFeB layer with a coercive field of H_c_ = 1.7 ± 0.2 mT. The spin-up and spin-down states can be inferred from the high and low Hall resistance states, respectively, as shown schematically in the insets of Fig. 1(d). Using vibrating sample magnetometry (VSM), we then measured the magnetization hysteresis loops for the bottom CoFeB layer within the field range of ±5 mT along the x-axis and y-axis, respectively, as shown in Fig. 1(e). Since the anisotropy field for the top PMA CoFeB layer exceeds hundreds of mT^5^, the magnetization contribution in Fig. 1(e) from the top layer is negligible within ±5 mT. Figure 1(e) indicates that the x-axis and y-axis are the hard and easy axis, respectively, for the bottom CoFeB layer. The measured uniaxial magnetic anisotropy field H_k_ (or the saturation field) is 2.5 ± 0.1 mT, and the H_c_ is 0.45 ± 0.05 mT. H_K_ is much larger than H_C_ by 5.6-fold, which is evidence of the domain-nucleation assisted switching process for the bottom CoFeB layer. We estimate that the uniaxial magnetic anisotropy constant, ${K}_{u}=\frac{1}{2}{H}_{K}{M}_{S}$, is about 1.06 $\times {10}^{4}\,\mathrm{erg}/{{\rm{cm}}}^{3}$ for the 3-nm-thick CoFeB layer.

## Current Induced Magnetic Switching and Critical Switching Current Density

In order to manipulate the magnetization state ($\mathop{{\rm{M}}}\limits^{\rightharpoonup }$) of the active element, which is the top PMA CoFeB(1.0) layer in Fig. 1(b), we need to exert a net torque (${\mathop{{\rm{\tau }}}\limits^{\rightharpoonup }}_{{\rm{TOT}}}$) onto the $\mathop{{\rm{M}}}\limits^{\rightharpoonup }$ vector. ${\mathop{{\rm{\tau }}}\limits^{\rightharpoonup }}_{{\rm{TOT}}}$ consists of three components^5,7,21^: the spin-transfer torque ${\mathop{\tau }\limits^{\rightharpoonup }}_{st}$ arising from the spin current generated by β-W; the external torque ${\mathop{{\rm{\tau }}}\limits^{\rightharpoonup }}_{ext}$ from external applied magnetic field ${\mathop{{\rm{B}}}\limits^{\rightharpoonup }}_{{\rm{ext}}}$ along the y-axis; and the anisotropy-field torque ${\mathop{\tau }\limits^{\rightharpoonup }}_{an}$ from the PMA. By projecting ${\mathop{{\rm{\tau }}}\limits^{\rightharpoonup }}_{{\rm{TOT}}}$ onto the x-axis, one obtains,

1 $${\tau }_{{\rm{T}}{\rm{O}}{\rm{T}}}=\mathop{{\rm{x}}}\limits^{\rightharpoonup }\cdot ({\mathop{\tau }\limits^{\rightharpoonup }}_{{\rm{s}}{\rm{t}}}+{\mathop{\tau }\limits^{\rightharpoonup }}_{{\rm{e}}{\rm{x}}{\rm{t}}}+{\mathop{\tau }\limits^{\rightharpoonup }}_{{\rm{a}}{\rm{n}}})={\tau }_{{\rm{s}}{\rm{t}}}^{0}+{{\rm{B}}}_{{\rm{e}}{\rm{x}}{\rm{t}}}\,\sin (\theta -\delta )-{{\rm{B}}}_{{\rm{a}}{\rm{n}}}^{0}\,\sin \,\theta \,\cos \,\theta $$

The spin-transfer torque ${\tau }_{{\rm{s}}{\rm{t}}}^{0}$ is used to switch $\mathop{{\rm{M}}}\limits^{\rightharpoonup }$ (up and down). During the switching process, an in-plane magnetic field ${{\rm{B}}}_{\text{ext}}$ is required to break the mirror symmetry with respect to the x-z plane, so that $\mathop{{\rm{M}}}\limits^{\rightharpoonup }$ rotates coherently in one direction (*e.g*., clockwise) under either a positive or negative current inside the GSHE β-W layer^7,12^.

**Figure 2:**
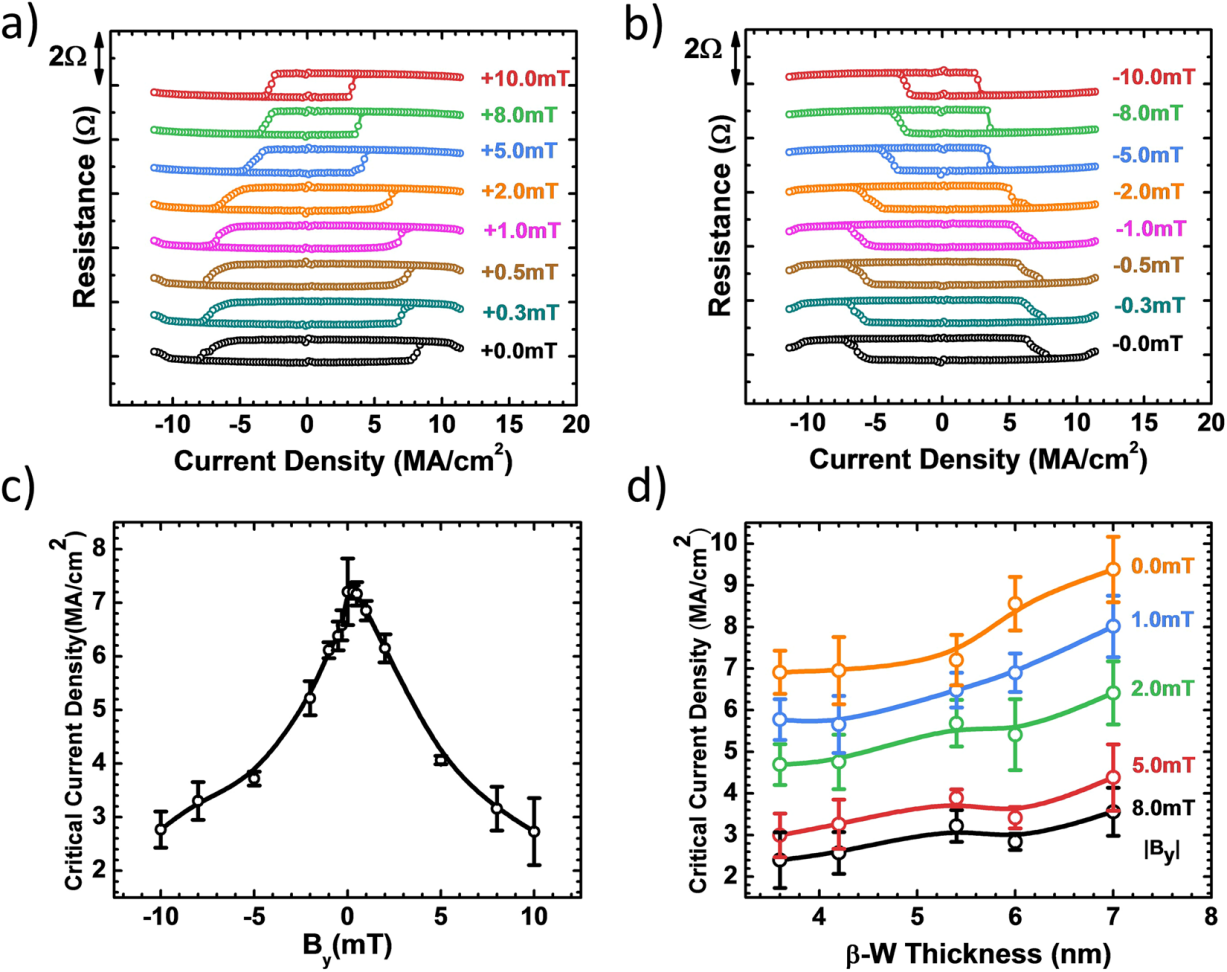
Current induced magnetic switching. (**a**,**b**) Hall resistance with cycling current under the external in-plane magnetic field from ±10 mT to ±0 mT. Both measurements were conducted with the sweeping current density from $-11.4\,{\rm{MA}}/{{\rm{cm}}}^{2}$ to $11.4\,{\rm{MA}}/{{\rm{cm}}}^{2}$ and then reverse to $-11.4\,{\rm{MA}}/{{\rm{cm}}}^{2}$ within one loop. (**c**) Critical switching current density of x = 5.4 nm sample versus different external magnetic field. Each point is averaged from 3 times repeated switching measurement as in (**a**,**b**). (**d**) Critical switching current density versus β-W thickness (3.6 nm~7.0 nm) and external magnetic biasing field.

Figure 2(a) shows the anomalous Hall resistance of our sample as a function of cycling charge current density (up to ±1.14 × 10^7^ A/cm^2^ or ±11.4 MA/cm^2^) within the β-W layer, as the sample is subjected to ${{\rm{B}}}_{\text{ext}}$ = +B_y_, ranging from +10.0 mT to 0.0 mT. For each given ${{\rm{B}}}_{\text{ext}}$ including ${{\rm{B}}}_{\text{ext}}$ = 0 (zero-field), well-defined magnetic switchings of the top CoFeB(1.0) magnetic state are clearly observed. Particularly noteworthy is the attainment of the field-free deterministic magnetic switching, a major requirement to make reliable magnetic memory or spin-logic operations palatable based on the GSHE-enabled technology. The presence of ${{\rm{B}}}_{\text{ext}}$ does reduce the critical switching current density (J_c_), which ranges from $7.2\pm 0.6\,{\rm{MA}}/{{\rm{cm}}}^{2}$ at field-free to $2.8\pm 0.3\,{\rm{MA}}/{{\rm{cm}}}^{2}$ at 10 mT. These values of J_c_ are among the lowest observed in similar field-free switching systems^12–17^, due to the fact that β-W has the highest spin Hall angle^4,8,10,19^, *i.e*., the highest efficiency of charge to spin current conversion. Figure 2(b) is similar to Fig. 2(a), except ${{\rm{B}}}_{\text{ext}}$ = −B_y_, *i.e*., under a negative external field.

Figure 2(c) shows the effect of bipolar ${{\rm{B}}}_{\text{ext}}$ = ±B_y_ on critical switching density J_c_, which is symmetrical with respect to the field-free case. We repeated the same magnetic switching measurement as in Fig. 2(a,b) at least 3 times to obtain the average J_c_. We also studied the effect of β-W thickness on J_c_ within the range of 3.6 nm ≤ x ≤ 7.0 nm. Field-free deterministic switching is realized in every case, with results similar to Fig. 2(a,b and c). We summarize the results in Fig. 2(d). As thickness x increases within the range studied, the field-free J_c_ value is 6.9 ± 0.5 MA/${{\rm{cm}}}^{2}$ at x = 3.6 nm and 9.4 ± 0.8 MA/${{\rm{cm}}}^{2}$ at x = 7.0 nm.

## Pulse Current Induced Magnetic Switching

**Figure 3:**
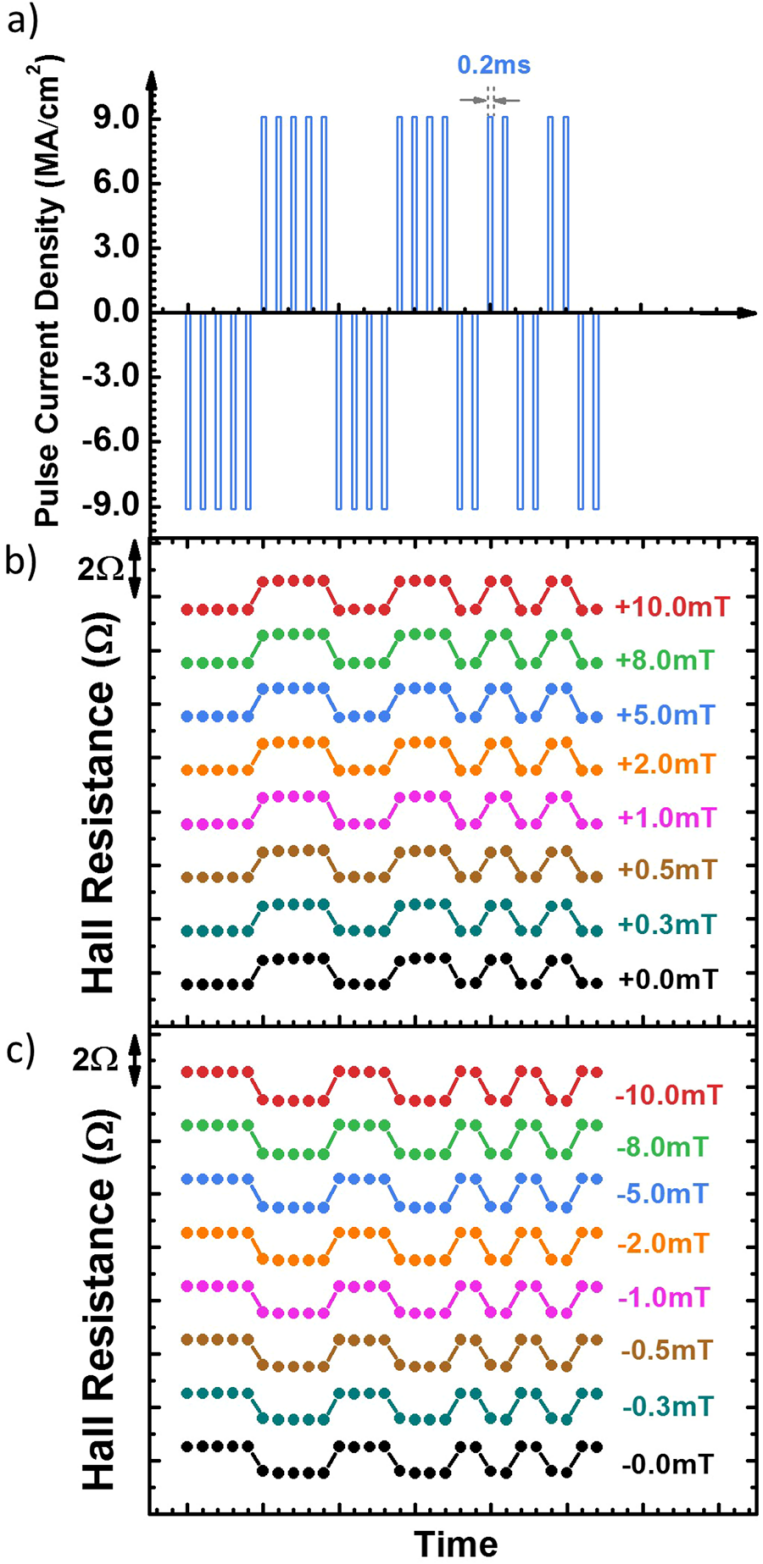
Pulse current induced magnetic switching. (**a**) A time sequence of current pulses with an amplitude of $9.0\,\text{MA}/{{\rm{cm}}}^{2}$ and a width of 0.2 ms. (figure is not to scale). Measurement is conducted during the pulse. (**b**,**c**) Hall resistance response under the pulse current sequence in a) under the external magnetic field from ±10.0 mT to ±0.0 mT.

We tested the consistency of current induced switching by applying bipolar square current pulses to the samples with arbitrary sequences. Figure 3(a) shows a representative and specific time sequence of current pulses, with an amplitude of $9.0\,\text{MA}/{{\rm{cm}}}^{2}$ and a width of 0.2 ms per pulse. Figure 3(b) shows the binary spin states (up and down, as inferred from Hall resistance) of the top CoFeB layer, controlled by the current pulse train for a set of biasing fields from +10.0 mT to field-free, with the bottom CoFeB layer spin state always along the y-axis. In every case with or without an external biasing field, the spin states respond to the bipolar current pulses consistently and repeatedly. In other words, the spin-down state is switched to the spin-up state upon the application of a positive current pulse, and vice versa. Symmetrically, we performed the same experiment under the same set of biasing fields, with the opposite sign, *i.e*., from −10.0 mT to field-free, as shown in Fig. 3(c). In this case, the bottom layer spin state is always along the negative y-axis (right to left). Consequently, we observed a reversed response of the spin states to the current pulse train. That is, the spin-down state was switched to the spin-up state upon the application of a negative current pulse, and vice versa. Visually, the response shown in Fig. 3(c) is anti-symmetrical to that shown in Fig. 3(b), exclusively due to the direction of the biasing field (external or internal). In the case of field-free (0 mT), the bi-stable spin-states of the bottom layer, as shown by the square loop in Fig. 1(e), are responsible for the existence of a positive and a negative biasing field.

## Discussion

As shown above, we have demonstrated current induced magnetic switching by virtue of GSHE of β-W without the need of an external magnetic field. However, a local in-plane biasing field is required for such switching. We estimate the effective field in our sample based on a commonly-acknowledged fact: The relationship between critical current density and the external field is one-to-one mapping. The larger external field corresponds to the smaller critical current density. In Fig. 2(d), the critical current density at zero field is 6.9~9.4 MA/cm^2^ for 3.6~7.0 nm β-W samples. Comparing with previously published study about critical current density under various external field in the similar structure, we conclude that the magnitude of the effective field is in the range of 0.2~0.5 mT^5,6,8^. The question arises: What provides such internal field at zero external field to achieve switching. The key to the answer is the 3 nm-thick CoFeB layer; otherwise, the field-free switching is impossible without this layer. We can rule out the Oersted field effect of charge current, since Oersted field is smaller than 0.05 mT using COMSOL Multiphysics^®^ simulation and exists independently with the bottom CoFeB layer. In the following parts, we will evaluate quantitatively the physical mechanisms from the 3 nm-thick CoFeB layer and their contribution to effective field.

**Figure 4:**
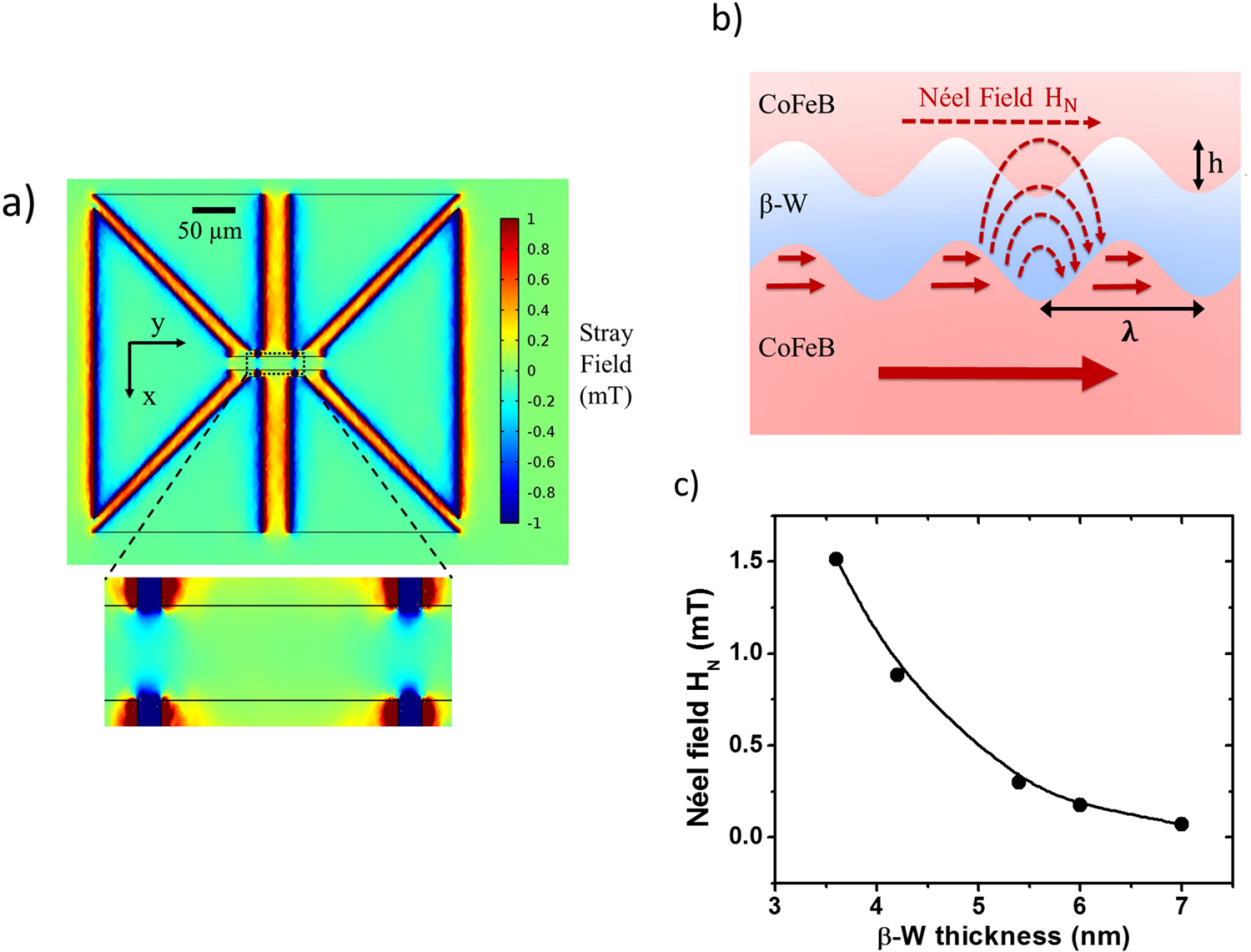
The Stray field and Néel field from 3 nm-thick CoFeB layer. (**a**) Simulation result of y-component distribution of the stray field at 1 nm-thick CoFeB layer using COMSOL Multiphysics^®^. (**b**) Schematic cross-section view of a sinusoidal-like interface roughness profile in the structure as CoFeB/β-W/CoFeB. (**c**) Semi-log of simulated Néel field versus various β-W thickness.

We calculate the stray field, which emanates from the bottom 3 nm-thick CoFeB layer, using COMSOL Multiphysics^®^ simulation software. Figure 4(a) shows the simulated in-plane y-axis component of the stray field distribution on the top CoFeB layer over the entire Hall bar sample of Fig. 1(c) (based on the CoFeB magnetization of 848 emu/cm^3^). The area of interest regarding magnetic switching is enlarged in Fig. 4(a). Two features are observed in this figure. Firstly, over the main area, the magnitude of the stray field is on the order of 0.1 mT, which is insufficient to support zero ambient field switching. Secondly, the stray field is about 0.5 mT in the vicinity of the voltage leads. It is possible that this stray field near the voltage leads might assist the magnetic switching through the domain-nucleation, but such switching is non-deterministic since the y-component of the stray field is either positive or negative. Based on these two facts, we believe that stray field cannot be the mechanism for zero-field switching in our samples.

The second possible mechanism is the Néel “orange-peel” effect, which has been well studied in magnetic tunneling junctions (MTJs). It arises from the proximity of the two magnetic layers separated by a non-magnetic layer^22,23^. Figure 4(b) shows a schematic cross-section view of a sinusoidal-like interface roughness profile and the resulting Néel field. The direction of Néel field is the same as the magnetization direction of the bottom 3 nm-thick CoFeB layer, which is consistent with the required biasing field for magnetic switching. The magnitude of the Néel field is given by

2 $${{\rm{H}}}_{{\rm{N}}}=\frac{{{\rm{\pi }}}^{2}}{\sqrt{2}}(\frac{{{\rm{h}}}^{2}}{{{\rm{\lambda }}t}_{{\rm{FM}}}}){{\rm{M}}}_{{\rm{S}}}{{\rm{e}}}^{-2\sqrt{2}{{\rm{\pi }}t}_{{\rm{NM}}}/{\rm{\lambda }}}$$

where ${{\rm{t}}}_{{\rm{FM}}}$ and ${{\rm{t}}}_{{\rm{NM}}}$ are the thickness of the top FM layer (1 nm in our samples) and the NM layer, respectively; h and λ are the peak-to-peak amplitude and the wavelength of the sinusoidal roughness profile as illustrated in Fig. 4(b); and ${{\rm{M}}}_{{\rm{S}}}$ is the saturated magnetization of the top FM layer. Using Eq. (2) and parameters pertinent to our samples, we have calculated ${{\rm{H}}}_{{\rm{N}}}$ as a function of β-W thickness, as shown in Fig. 4(c). The Néel field decays exponentially with the increasing β-W thickness in the range of our samples as presented in Fig. 2(d) from 3.6 nm to 7.0 nm. ${{\rm{H}}}_{{\rm{N}}}$ is as high as 1.5 mT for a 3.6 nm β-W, and it decreases to 0.3 mT for 5.4 nm β-W, to 0.08 mT for 7 nm β-W. The parameters of h and λ in Eq. (2) are taken from the analysis of cross-section transmission electron micrographs of similarly fabricated multilayer structures by our research group^22–24^. Figure 4(c) provides us with a reasonable estimation of the Néel field in our samples.

The third possible mechanism is the indirect interlayer exchange coupling effect. This coupling effect in the FM/NM/FM structure has been widely investigated^25–29^, where FM are Fe, Co, Ni, or ferromagnetic alloys, and NM are 3d, 4d, or 5d transition non-magnetic metals. The interlayer exchange is often long-range and oscillatory in polarity (antiferromagnetic to ferromagnetic), caused by the Fermi surface topology of a NM^25–28^. This type of structure has been implemented in spin-valve GMR (giant magnetoresistance) sensors^30,31^ or in MTJs^32,33^. In our unique structure which can be labeled as CoFeB(→)/β-W/CoFeB(↑), we have used β-W both as a GSHE agent and as a conducting metallic solid for mediating interlayer exchange between an in-plane FM layer and a PMA FM layer. The interlayer exchange interaction creates a small tilt on the “vertical” spin-state of the top layer, therefore, breaking the x-z plane symmetry (see Fig. 1(a)). In effect, the interlayer exchange behaves like a y-axis biasing field as far as the top layer is concerned. In the β-W thickness range of 3.6 to 7.0 nm that we studied, the interlayer exchange coupling is always ferromagnetic, as concluded from our measurement. The interlayer exchange coupling through β-W has not been investigated in the literature, although there is data available^26^ for Co/α-W/Co and Fe/α-W/Fe. While α-W is a bcc solid with a lattice constant of 0.3165 nm, β-W is an *A*_*3*_*B* solid with the *A*15 type crystal structure (lattice constant 0.504 nm)^20^. The maximum antiferromagnetic coupling was found to be on the order of 0.03 erg/cm^2^, occurring at an α-W thickness of 0.55 nm^26^. In comparison, Ru mediated coupling is on the order of 5 erg/cm^2^ ^26^. In our case, β-W thickness is extremely long-range (3.6~7.0 nm). Even so, a small ferromagnetic coupling is still possible to be realized in our structure CoFeB(→)/β-W/CoFeB(↑).

In addition to the three mechanisms generating an effective internal field discussed above, the so-called unconventional spin polarization, generated from the interface of CoFeB(→)/β-W, may be another possible mechanism of breaking the symmetry in the switching system presented in this article. The very recent studies^34,35^ propose a novel spin polarization of spin current from the FM/NM interface, which is different from the conventional GSHE. Baek *et al*.^34^ demonstrated experimentally that the spin current, generated from the FM(→)/NM interface, has an out-of-plane component of the spin polarization due to the net propagation of non-equilibrium carriers and the interfacial scattering effect. Humphries *et al*.^35^ observed the spin current with arbitrary polarization by manipulating the magnetization direction, which arises from a combination of spin rotation symmetry and spin obit effect symmetry in FM/NM structure. Such spin polarization is beneficial to the switching of the PMA layer at zero field^12,34,35^.

Based on our analysis above, we believe that field-free switching arises mainly from Néel field and interlayer exchange coupling. The Néel field in the samples (β-W thickness ≤5.4 nm) provides an unneglectable biasing field (greater than 0.3mT) in the preferred direction for zero-field switching. This mechanism can be certainly taken advantage of for zero ambient field switching. However, in the 7 nm β-W sample, the Néel field is estimated as 0.08 mT, which cannot account for the observed switching by itself. Additionally, the strength of interlayer exchange coupling is given by ${\rm{J}}={{\rm{H}}}_{{\rm{S}}}{{\rm{Mt}}}_{{\rm{F}}}/2$, where ${{\rm{H}}}_{{\rm{S}}},\,M,{t}_{{\rm{F}}}$ are the saturation magnetic field, magnetization and layer thickness of the ferromagnetic layer, respectively. For CoFeB(→)/β-W/CoFeB(↑), we conservatively estimate that ${\rm{J}}$ is 0.001~0.003 erg/cm^2^ ^26^. Feeding such value into interlayer exchange coupling, the saturation field is estimated as 0.7~2.1 mT, which is sufficient to break the y-z plane symmetry for the top PMA CoFeB layer. Besides, the apparent increase of critical switching current density with thickness, especially at zero field, indicates the decrease of the effective field with increasing β-W thickness and supports indirectly Néel field and interlayer exchange coupling. Finally, the so-called unconventional spin polarization, generated from the interface of CoFeB(→)/β-W, may be another possible mechanism for the observed switching in this article.

## Conclusion

We have developed a new method to achieve field-free deterministic current induced magnetic switching of a thin CoFeB film with bi-stable spin states enabled by the PMA. We use β-W film to generate a highly spin polarized current based on its Giant Spin Hall Effect. The large spin-transfer torque generated by the β-W film leads to a low critical switching current density of about $6.9\sim 9.4{\rm{M}}{\rm{A}}/{{\rm{c}}{\rm{m}}}^{2}$ under field-free condition. More importantly, we use the same β-W film as spacer layer between an in-plane 3 nm-thick CoFeB magnetic layer at the bottom and a switchable 1 nm-thick CoFeB layer with PMA at the top to achieve field-free switching. We estimated the several possible physical mechanisms that may be at play, including Néel “orange-peel” coupling effect, the stray field, interlayer exchange coupling, and unconventional spin polarization. Both the Néel and exchange coupling mechanisms are beneficial to zero ambient field switching, whereas the stray field lacks the strength as a mechanism. Overall, our method is simple, effective, and optimizable. It removes a major hurdle in the applications of magnetic memory and spin-logic devices.

## Methods

### Sample Preparation

We used a home-made magnetron sputtering system to deposit the multilayer samples on 2 inches thermally oxidized silicon wafers. The base pressure before sputtering was around 2 $\times {10}^{-8}$ Torr. During deposition, the substrate rotated at the speed of 50 rpm to ensure thickness uniformity. The multilayer structure was grown in one step without breaking vacuum condition. The multilayer stack is sequenced as follows: Si/SiO_2_/Ta(1.0)/CoFeB(3.0)/β-W(x)/CoFeB(1.0)/MgO(1.6)/Ta(1.0) (numbers in the parenthesis are thickness of corresponding layer in nanometers). We sputtered MgO using RF power under the 1.2 mTorr Ar pressure and sputtered all other materials using DC power under 2.2 mTorr Ar pressure. After deposition, we patterned our samples into 20 μm × 55 μm standard Hall bars by photolithography and ion milling. Finally, the patterned samples are annealed at 280 °C for 1 hour in the vacuum of 2 $\times {10}^{-6}$ Torr under the external in-plane magnetic field of 0.42 T.

### Sample Characterization

To calibrate the sputtering rate of each material, we used two instruments: Dektak^®^ Profilometer and Bruker D8 Discover^®^ X-ray Diffractometer. For the characterization of PMA, we measured Hall resistance versus perpendicular magnetic field, see Fig. 1(d), using Keithley 2400 Source Meter and Keithley 199 Scanner Multimeter. For magnetic characterization, we used Quantum Design^®^ vibrating sample magnetometer (VSM) (as a part of QD’s Physical Property Measurement System) to measure magnetization hysteresis loops, see Fig. 1(e), which gave us information about the 3 nm-thick CoFeB layer.

### Experimental Measurement

We utilized an Keithley 6221/2182A Source Meter/Nanovoltmeter combination to generate current pulse sequence and measure Hall resistance simultaneously. The current pulse width was 0.2 ms. The magnetic field, especially the zero field, was calibrated carefully using the Lakeshore 450 Gaussmeter exactly in the sample holder position. The uncertainty of magnetic field was less than 0.03 mT (smaller than the earth’s magnetic field). All the measurements in this paper were conducted at room temperature.

To evaluate the current density in the β-W layer, it is assumed the current flowing through the multilayer sample obeys the parallel resistance law. According to our previous study^5^, the resistivity of CoFeB and Ta layer in the similar annealed system are 100 µΩ-cm and 200 µΩ-cm, respectively.

#### Simulation

For Néel “orange-peel” coupling field, the parameters in the Néel model are obtained from the data and TEM images in the literature. The parameters for Néel field estimation in Fig. 4(c) are listed as follow: h = 8.0 Å, λ = 98 Å, t_FM_ = 1 nm, t_s_ = x nm, M_s_ = 848 emu/cm^3^.

We simulated the stray field using COMSOL Multiphysics^®^ in Fig. 4(a). The dimension of the hall bar device is the same as our real samples. The saturation magnetization is 848 emu/cm^3^ according to our VSM measurement.
